# Dilemma of ST-Segment Elevation and Subarachnoid Hemorrhage: A Case Report and Literature Review

**DOI:** 10.7759/cureus.70564

**Published:** 2024-09-30

**Authors:** Ghulam Mujtaba Ghumman, Nagabindhu Aravapalli, Fnu Salman, Mohammed Taleb, Sohail S Ali

**Affiliations:** 1 Cardiovascular Disease, Mercy Health - St. Vincent Medical Center, Toledo, USA; 2 Internal Medicine, Mercy Health - St. Vincent Medical Center, Toledo, USA

**Keywords:** cardiac arrest, hemorrhage, stemi, subarachnoid, vasospasm

## Abstract

Acute ST-segment elevation myocardial infarction (STEMI) occurs due to occlusion of one or more coronary arteries causing myocardial injury. It is a medical emergency and requires prompt diagnosis and intervention. Transient ST-segment elevation can occur due to coronary vasospasm, and their association has been reported with subarachnoid hemorrhage. We present a distinct case of ST-segment elevations in inferior leads with reciprocal ST-depressions in lateral leads, indicating STEMI that leads to complete heart block and ventricular fibrillation cardiac arrest in a patient with subarachnoid hemorrhage. The coronary angiogram was negative for any obstructive coronary artery disease.

## Introduction

Neurogenic stunned myocardium is a well-known entity in the medical literature. Subarachnoid hemorrhage (SAH) is one of the common neurological conditions that exhibit cardiac manifestations [1,2]. Various electrocardiogram (ECG) abnormalities are found in up to 62% of patients in SAH [3]. Often, these patients present with overlapping neurologic and cardiac manifestations, leading to clinical dilemmas and misdiagnosis, which can lead to delays in appropriate care. Very few cases have been reported in the literature of patients with SAH leading to ST-segment elevation myocardial infarction (STEMI) [1,4,5]. Almost all of the cases reported initially presented with cardiac manifestation and were later found to have neurological insults. We present a distinctive case of a 47-year-old female who was initially admitted for the management of traumatic SAH. She later developed transient inferior leads ST-segment elevations and complete heart block, leading to ventricular fibrillation cardiac arrest, with coronary angiogram showing normal coronaries.

## Case presentation

A 47-year-old female with no significant prior medical history and no chronic medication use presented to the emergency room following a fall after alcohol intoxication. According to family members, the patient and her brother had consumed alcohol, and after her brother dropped her off at home, she fell on her front steps, resulting in a head injury. Emergency medical services (EMS) were called, and at the scene, the patient was moaning and unable to follow commands but was protecting the airway. Upon arrival at the emergency department (ED), the vital signs were stable, with a blood pressure of 138/83 mmHg, heart rate of 65 beats/minute, respiratory rate of 16 breaths/minute, and temperature of 96.6°F. However, while in the ED, the patient became increasingly lethargic and was later intubated to protect the airway. A physical examination revealed a laceration to the left eyebrow, but it was otherwise unremarkable. A head computed tomography (CT) scan revealed a small SAH without any mass effect or midline shift. A comprehensive trauma workup included whole spine CT scans and CT scans of the chest, abdomen, and pelvis, all of which yielded negative results. Initial laboratory tests showed normal blood cell counts, normal electrolyte levels, and normal renal function. The urine toxicology screen was negative for any drug use. 

**Figure 1:**
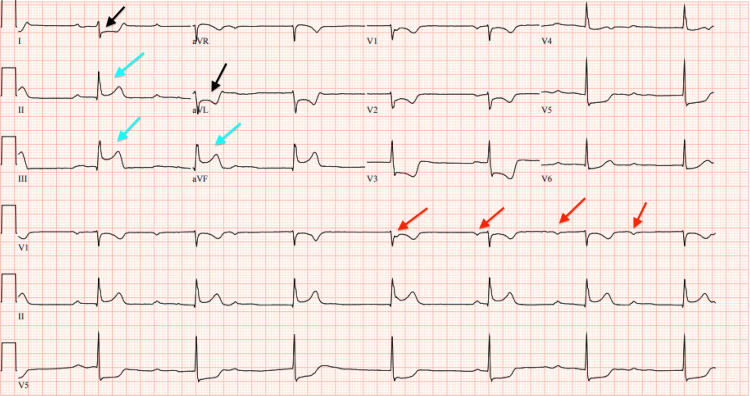
Electrocardiogram (ECG) showing third-degree atrioventricular (AV) block (marching P-waves marked by red arrow), ST elevations in leads II, III, and augmented vector foot (aVF) (blue arrows), with reciprocal changes in leads I and augmented vector left (aVL) (black arrows).

The patient was subsequently admitted to the trauma intensive care unit (ICU) for further care. On the second day of hospitalization, the patient developed hypotension and bradycardia with a heart rate in the 40s. ECG demonstrated third-degree atrioventricular block with ST-segment elevation in leads II, III, and augmented vector foot (aVF), along with reciprocal changes in lead I and augmented vector left (aVL), indicating ST-segment elevation myocardial infarction (STEMI) (Figure 1).

**Figure 2:**
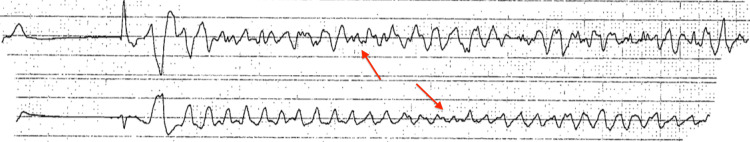
Rhythm strip showing ventricular fibrillation (red arrows).

Within minutes, the patient experienced a ventricular fibrillation cardiac arrest requiring one round of CPR with a return of spontaneous circulation (ROSC) without the need for defibrillation (Figure 2).

**Figure 3:**
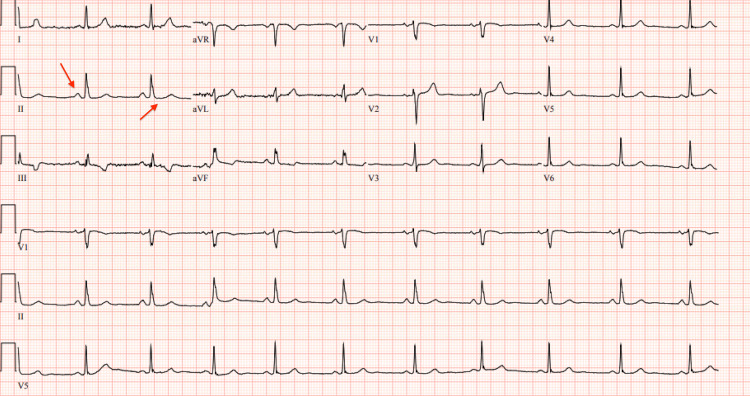
Post-cardiac arrest electrocardiogram (ECG) showing normal sinus rhythm without any ST changes (red arrows).

The post-ROSC ECG revealed a normal sinus rhythm without any ST elevations, which were observed on the initial ECG (Figure 3).

Due to the concurrent SAH and the resolution of the ST changes, the cardiology and neurosurgery teams decided not to pursue immediate cardiac interventions. A repeat head CT showed that SAH was stable. The patient was extubated the following day.

**Figure 4:**
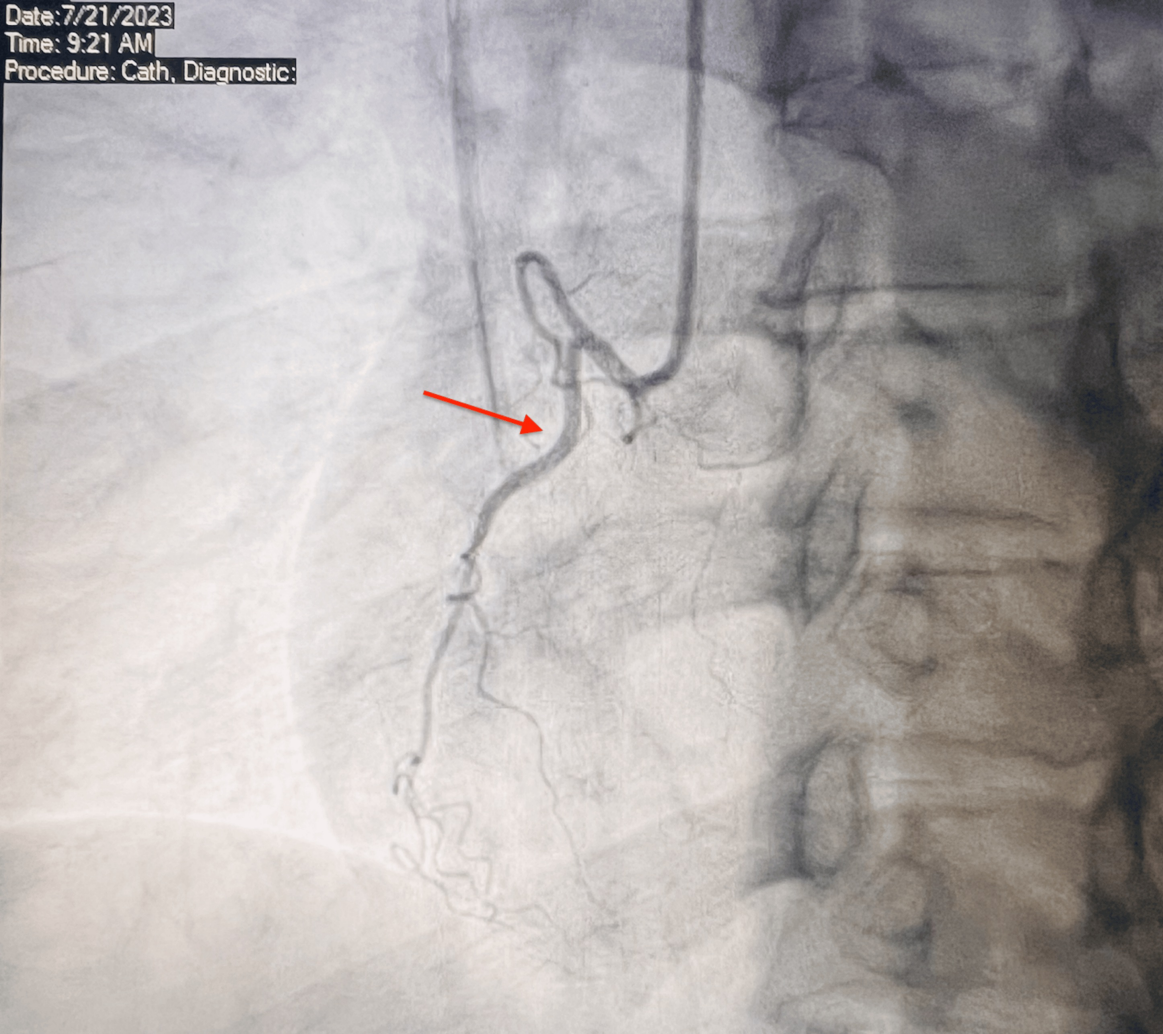
Coronary angiogram showing small non-dominant right coronary artery (red arrow).

**Figure 5:**
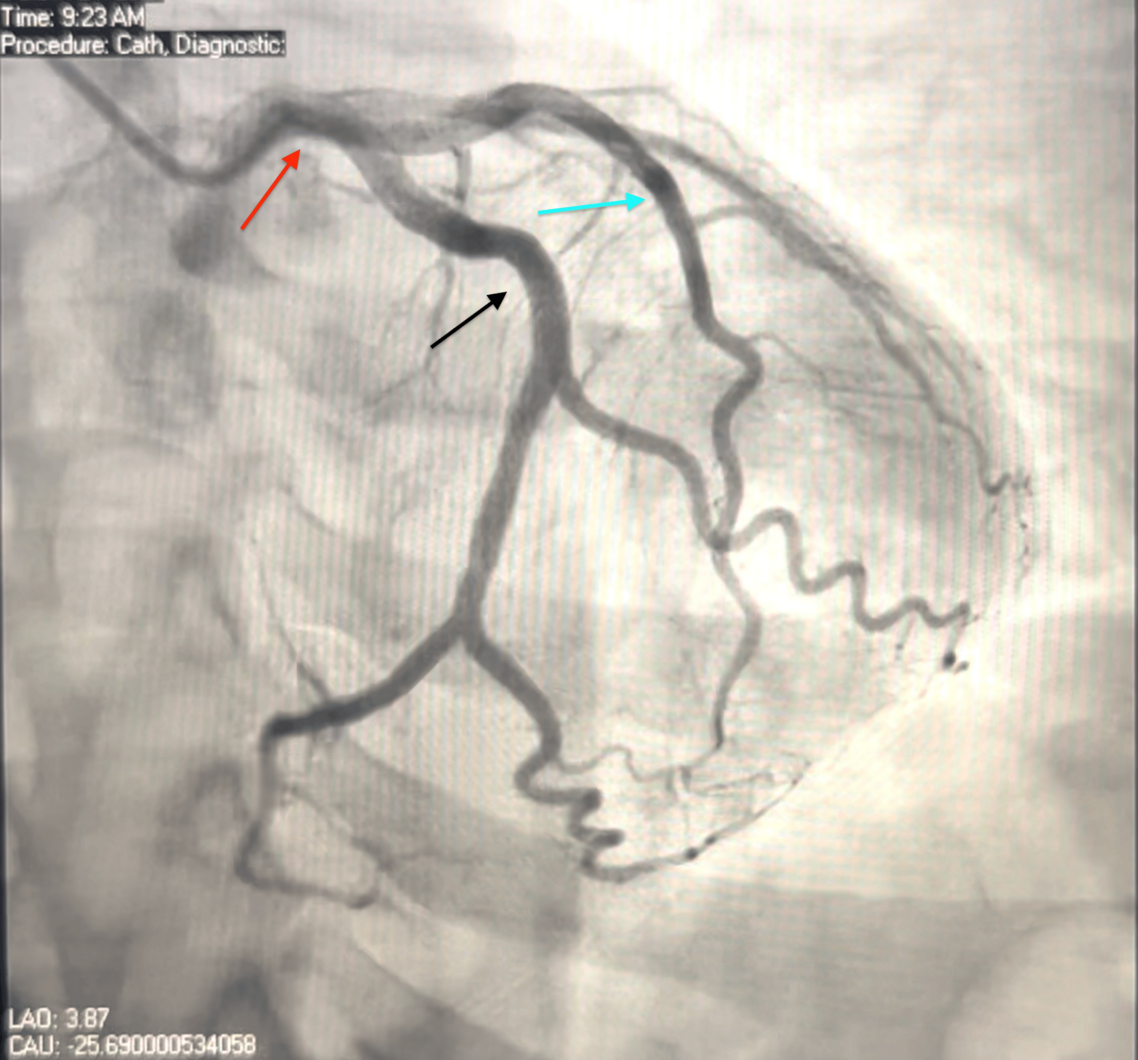
Coronary angiogram showing left dominant circulation with patent left main (red arrow), left anterior descending (blue arrow), and left circumflex (black arrow) arteries.

Considering the potential for SAH-induced cardiac injury, a CT coronary angiogram was performed, but the results were inconclusive. The echocardiogram revealed an ejection fraction (EF) of 60-65% with no wall motion abnormalities. On the sixth day of hospitalization, the patient underwent cardiac catheterization, which showed normal coronary arteries and normal left ventricular (LV) function (Figure 4 and Figure 5).

Subsequently, an implantable cardioverter-defibrillator (ICD) was placed by the electrophysiology team for secondary prevention, and the patient was discharged with plans for outpatient follow-up with the cardiology team. At the cardiology outpatient visit, the patient reported no complaints and was in good health.

## Discussion

SAH can exhibit various cardiac manifestations, spanning ST-segment deviations, T wave inversions, heart blocks, prolonged QT intervals, elevated cardiac biomarkers, and LV dysfunction despite unobstructed coronary arteries [6,7]. While ECG changes frequently occur in cases of SAH, the occurrence of typical myocardial ischemic changes is uncommon, and instances of typical ST-segment elevations are even rarer. A recent 2022 study investigating aneurysmal SAH in adults estimated the incidence of STEMI at 1.04% [8]. Nevertheless, reports of STEMI cases associated with traumatic SAH could not be identified during our literature review. Our case is a unique presentation of traumatic SAH complicated by ST segment elevations, complete heart block, and ventricular fibrillation cardiac arrest despite normal coronary arteries. 

The predominantly theorized pathophysiological mechanism involves an excess release of catecholamines triggered by neurogenic injury, resulting in transient coronary vasospasm, myocardial injury, and dysfunction [9]. ST-segment elevation in the acute stage of SAH reflects transient cardiac dysfunction rather than myocardial injury [10]. 

When SAH is associated with STEMI, the ST elevations are not frequently accompanied by the usual reciprocal changes [11,12]. In our presented case, distinct reciprocal changes in the lateral leads were manifested. It is also often observed that patients with SAH experience cardiac arrest in the form of pulseless electrical activity/asystole [12]. In contrast, our case developed STEMI along with complete heart block and cardiac arrest in the form of ventricular arrhythmia. These findings collectively suggested the presence of atherosclerotic coronary artery disease. However, the coronary angiogram showed normal coronary arteries. Although scant, there have been cases reported with concomitant STEMI and SAH, necessitating percutaneous coronary intervention with stenting [13,14]. Managing such complex cases entails intricate decisions across various medical specialties. Administering antiplatelet and anticoagulant treatments while contending with an active intracranial bleed poses a formidable challenge, demanding vigilant monitoring and thoughtful decision-making.

Cardiac injury resulting from SAH is linked to a heightened risk of future cardiovascular events and mortality, especially in patients with ST-T wave abnormalities [15]. Therefore, it is imperative to closely monitor these patients through regular follow-up appointments to identify any latent cardiac conditions and mitigate potential complications.

Through this case report, we contribute a unique presentation of the intersection between SAH and STEMI to the medical literature. Further research is imperative for better understanding the underlying pathophysiology and developing more effective management strategies for cardiac complications arising from neurological insults.

## Conclusions

In conclusion, this case report underscores the infrequent occurrence of neurogenic stunned myocardium within the context of traumatic SAH. While SAH frequently presents with various cardiac manifestations, developing a STEMI is notably rare. Our case, characterized by complete heart block, ST elevations on ECG indicating STEMI, and ventricular fibrillation, remarkably revealed normal coronary arteries. This report contributes a valuable perspective to the interplay between SAH and STEMI, emphasizing the necessity for further research to enhance our understanding of the underlying pathophysiology and develop more effective management strategies for neurological insult-induced cardiac complications.
